# Determinants and Experiences of Repeat Pregnancy among HIV-Positive Kenyan Women—A Mixed-Methods Analysis

**DOI:** 10.1371/journal.pone.0131163

**Published:** 2015-06-29

**Authors:** Victor Akelo, Eleanor McLellan-Lemal, Lauren Toledo, Sonali Girde, Craig B. Borkowf, Laura Ward, Kenneth Ondenge, Richard Ndivo, Shirley L. Lecher, Lisa A. Mills, Timothy K. Thomas

**Affiliations:** 1 Kenya Medical Research Institute, Kisumu, Kenya; 2 Centers for Disease Control and Prevention, Atlanta, GA, United States of America; 3 ICF International, Atlanta, GA, United States of America; 4 Rollins School of Public Health, Emory University, Atlanta, GA, United States of America; 5 Centers for Disease Control and Prevention, Kisumu, Kenya; University of Missouri-Kansas City, UNITED STATES

## Abstract

**Objective:**

To identify factors associated with repeat pregnancy subsequent to an index pregnancy among women living with HIV (WLWH) in western Kenya who were enrolled in a 24-month phase-II clinical trial of triple-ART prophylaxis for prevention of mother-to-child transmission, and to contextualize social and cultural influences on WLWH’s reproductive decision making.

**Methods:**

A mixed-methods approach was used to examine repeat pregnancy within a 24 month period after birth. Counselor-administered questionnaires were collected from 500 WLWH. Forty women (22 with a repeat pregnancy; 18 with no repeat pregnancy) were purposively selected for a qualitative interview (QI). Simple and multiple logistic regression analyses were performed for quantitative data. Thematic coding and saliency analysis were undertaken for qualitative data.

**Results:**

Eighty-eight (17.6%) women had a repeat pregnancy. Median maternal age was 23 years (range 15-43 years) and median gestational age at enrollment was 34 weeks. In multiple logistic regression analyses, living in the same compound with a husband (adjusted odds ratio (AOR): 2.33; 95% confidence interval (CI): 1.14, 4.75) was associated with increased odds of repeat pregnancy (p ≤ 0.05). Being in the 30-43 age group (AOR: 0.25; 95% CI: 0.07, 0.87), having talked to a partner about family planning (FP) use (AOR: 0.53; 95% CI: 0.29, 0.98), and prior usage of FP (AOR: 0.45; 95% CI: 0.25, 0.82) were associated with a decrease in odds of repeat pregnancy. QI findings centered on concerns about modern contraception methods (side effects and views that they ‘ruined the womb’) and a desire to have the right number of children. Religious leaders, family, and the broader community were viewed as reinforcing cultural expectations for married women to have children. Repeat pregnancy was commonly attributed to contraception failure or to lack of knowledge about post-delivery fertility.

**Conclusions:**

In addition to cultural context, reproductive health programs for WLWH may need to address issues related to living circumstances and the possibility that reproductive-decision making may extend beyond the woman and her partner.

## Introduction

HIV prevalence has remained disproportionately higher among females in sub-Saharan Africa, who constitute 92% of all HIV-positive pregnant women globally [1]. In sub-Saharan Africa, young women aged 15–24 years are eight times more likely than men of the same age to be HIV infected [2]. In Kenya, HIV prevalence among women aged 15–64 years is 8.2% compared to 6.4% prevalence rate among men in the same age group [3]. Kenyan women of reproductive age have an even higher HIV prevalence of 10% and 11% among 25–29 year olds and 30–39 year olds, respectively [3]. Although there is increased use of contraceptives by women living with HIV (WLWH), pregnancies still occur [4].

In India, the United Kingdom, and Ireland, repeat pregnancy among HIV-infected women in HIV care increased from approximately 20% to 39% between 1997 and 2009 [4, 5]. This increase has been attributed mainly to an improved maternal survival rate as a result of antiretroviral therapy (ART), increased fertility among HIV-positive women given improved quality of life, and optimism about having HIV-uninfected children due to programs that reduce mother-to-child transmission [6–11]. A systematic review has shown that most HIV-positive women on ART remained healthy during pregnancy and that pregnancy did not increase HIV diseases progression [12]. However, the literature suggests that even in countries with a more advanced approach to HIV care, as few as 25% of HIV-positive female patients discuss childbearing with their health care providers [13].

HIV status alone has been shown to neither predict nor influence a woman’s child bearing intentions [14]. Pregnancy decisions among HIV-positive women are influenced by family members, healthcare providers, and economic circumstances [15]. A qualitative study among HIV-positive Kenyan women found that childbearing carries an important cultural meaning that serves to solidify marriage [16]. In a polygamous marriage, a wife’s influence in the home is leveraged by the number of children she has; hence, infertility may lead to marital disharmony and lower the wife’s social status [16]. Healthcare providers counsel WLWH to wait until they have improved their immunologic status and physical health before considering having children; yet most WLWH will have children sooner to secure their relationship with their partners, despite increased risk of maternal morbidity associated with shorter birth intervals [17].

Limited but increasing attention to reproductive decision making for WLWH in sub-Saharan Africa suggests that a complex set of clinical, psychosocial, religious, and cultural factors influence attitudes toward and experiences of repeat pregnancy. We undertook a mixed-methods study to examine factors associated with repeat pregnancy among WLWH enrolled in a prevention of mother-to-child transmission (PMTCT) HIV clinical trial. We hypothesize that reproductive decisions for WLWH extends beyond the known woman’s factors such as age, being in a relationship, partner’s desires, having a living child and being on ART [18–20] to include cultural, religious, psychosocial and familial factors. Qualitative interviews with a subset of participants were conducted to gain better insights into factors influencing reproductive decision making among WLWH. To the best of our knowledge only a few mixed-methods study have been conducted among WLWH to examine the complex interplay of familial, religious, psychosocial, cultural, and demographic factors associated with repeat pregnancy.

### Traditional Luo household structure

In the traditional societies of western Kenya, family units consist of parents, children, grandparents, uncles, aunts, brothers, sisters, and other relatives [21]. In a polygamous marriage (a common practice among the Luo, the largest ethnic group in Nyanza Province in western Kenya), a man usually provides each wife with a house of her own within the same compound where his other wives also live [21, 22]. A deep sense of kinship in the Kenyan community is fostered through blood ties and marriage customs [21, 22]. After marriage, a woman is expected to bear multiple children for her husband, which in turn enhances her standing and respect within the community [22]. A woman’s position and respect is further enhanced by her fertility: infertility could be used by the husband as a reason for obtaining another wife [23].

In the process of conducting an HIV PMTCT clinical trial (described below), we learned that a number of study participants, who had opted to use injectable depot medroxy-progesterone acetate (DMPA) after delivery, were not keeping appointments that had been arranged through local family planning referral facilities. In line with the Government of Kenya guidelines and practice, participants are encouraged to use a family planning method starting six weeks post delivery. Moreover, unanticipated pregnancies subsequent to the index pregnancy (i.e., repeat pregnancy during two-year post-partum follow-up) were observed. Consequently, a qualitative sub-study was conducted to better understand reproductive intents and decision making among WLWH.

## Methods

### Ethics statement

The Kisumu Breastfeeding Study (KiBS) was approved by the ethical review committees at the Kenya Medical Research Institute (KEMRI) and United States Centers for Disease Control and Prevention (CDC). KiBS clinical trial number is NCT00146380 [A Study of Zidovudine/Lamivudine and Either Nevirapine or Nelfinavir for Reduction of Mother-to-child HIV Transmission during Breastfeeding (KiBS)]. Written documentation of informed consent was provided by all research participants prior to undergoing any study procedures and data collection. For women younger than 18 years, a parent’s or legal guardian’s written consent and the participant’s assent were obtained.

### Study design and population

KiBS was a phase-II, open-label, one-arm PMTCT clinical trial with a 24-month follow-up in western Kenya. It assessed the effectiveness, safety, and tolerability of using maternal highly active antiretroviral combination therapy (HAART) to maximally suppress maternal viral load in the late antenatal period and during lactation with the aim of reducing mother-to-child transmission among breastfeeding HIV-infected women in a resource-limited setting. KiBS was conducted between July 2003 and February 2009. Study procedures and primary findings have been previously published [10].

KiBS recruitment was conducted through the antenatal clinics of the Kisumu District Hospital and Jaramogi Oginga Odinga Teaching and Referral Hospital (formerly known as Nyanza Provincial General Hospital). All women attending these clinics were offered voluntary counseling and HIV testing as a routine standard of care. To be eligible to participate in KiBS, a woman had to be at least 15 years of age, HIV infected, willing to initiate HAART, pregnant (between 34 and 36 weeks gestation), willing to receive counseling on and follow the UNAIDS breastfeeding guidelines, and planning to remain a resident of Kisumu for two years following study enrollment. Counseling on contraceptive methods for preventing unintended future pregnancies was provided at all study visits. Additional counseling was available for women who chose to disclose their HIV status to others, as well as for dealing with their families’ reaction to their family planning (FP) decisions. Women were encouraged to come with their male partner for couple-based counseling and HIV testing.

In total, 522 women enrolled in KiBS; 22 women were excluded from this analysis because they withdrew (21) or died (1) before delivery. Documentation of a subsequent pregnancy following the index KiBS pregnancy (henceforth referred to as the index pregnancy) was obtained through multiple sources (questionnaires, laboratory results, and home visits). Our study does not account for potentially missing documentation of a repeat pregnancy for participants lost to follow-up or having subsequent pregnancies after their 24 months of post-partum follow-up.

For the qualitative sub-study, a non-probabilistic, purposive sampling approach was used to select a subset of KiBS participants with and without a documented repeat pregnancy to take part in a semi-structured, open-ended interview. The sampling plan sought to interview 15 to 20 participants who had experienced a repeat pregnancy and another 15 to 20, participants who had not experienced a repeat pregnancy. A saturation approach, in which interview would continue to be collected until no new information was being provided, was used to determine the actual sample size. Debriefing sessions facilitated by a Masters-level anthropologist were conducted after each interview to discuss information heard and ascertain if saturation had been reached.

Women were eligible to take part in the sub-study if they were at least 18 years of age, had not exceeded more than 18-months of KiBS follow-up, and were willing to share their pregnancy-related experiences and cultural beliefs and attitudes around fertility. Women who had withdrawn their KiBS participation or who had completed more than 18 months of KIBS follow-up were not eligible for sub-study participation.

All interviews were completed using an open-ended, semi-structured interview guide. To minimize potential reluctance by study participants to share information regarding reproductive intents or concerns given possible concerns that might be negatively judged for not following the family planning counseling recommendations, neither the study clinicians nor the counselors participated in the data collection. Interviews were conducted by three staff members who were fluent in all three data collection languages. Two held Bachelor’s degrees in the social sciences and the third a national higher diploma in community development and project management. All interviewers completed training that emphasized *emic information-centered* qualitative interviewing skills and participated in a series interview role playing and interview debriefing exercises before conducting interviews.

All participants completed an audio-recorded interview in the clinic, their home, or another location of choice. After undergoing a sub-study-specific written informed consent process, an hour-long interview was conducted in English, Kiswahili, or Dholuo, based on the participant’s language preference.

### Data collection

#### Quantitative data

Study nurse counselors administered questionnaires in English, Kiswahili, or Dholuo (the predominant languages of the area) over the three-year enrollment period between July 2003 and November 2006. Our analyses focus on the following demographic, behavioral, and psychosocial measures collected at baseline: maternal age, average monthly income, marital status, highest level of education, intention to use FP, having talked to the partner about FP use, and living situation or co-residence; i.e., living with the child’s father, and/or other relatives in the compound (in-laws, parents, co-wives, and any other males). For the purposes of this study, we defined repeat pregnancy as a documented pregnancy occurring subsequent to the index pregnancy at enrollment and during the 24 months of post-partum follow up.

#### Qualitative data

All qualitative interviews were audio recorded and completed using an interview guide that consisted of questions on reproductive desires and intents after the birth of the index child; familial, cultural, and religious expectation on women’s roles; and communication between a woman and her partner. Women received a bar of soap at the completion of the interview and transportation reimbursement if the interview took place in a location other than their home.

### Data analyses

Using baseline data for all 500 participants who delivered on study, we present factors associated with having a ‘repeat’ pregnancy during the 24 months of post-partum follow-up, and then present findings from the 40 women who participated in the qualitative sub-study.

#### Quantitative data

Summary statistics (including percentages, medians, and ranges) were calculated for demographic, pregnancy history, and family planning variables. We also compared the proportions of women who had a repeat pregnancy by demographic and pregnancy intention variables using risk differences and chi-square tests. We performed simple and multiple predictor logistic regression analyses with repeat pregnancy as the outcome. To build the final model, we first performed tests for multicollinearity. We used variance inflation factors > 2 for pairwise comparisons as well as condition indices > 30 and variance decomposition proportions > 0.5 among three or more variables to indicate significant collinearity [24]. Next, we used stepwise selection methods to build a parsimonious multiple logistic regression (reduced) model using retention and entry criteria of p-value < 0.1 that also took into account the amount of missing data in candidate variables. The adequacy of the final model was assessed using the Hosmer-Lemeshow test (results not presented). Analyses were conducted using SAS software, version 9.3 (SAS Institute, Inc., Cary, NC, US).

#### Qualitative data

Audio-recordings were transcribed verbatim and non-English based transcripts translated into English for analysis. Thematic data analyses were performed using NVivo version 8 (QSR International Pty Ltd.). All text was read thoroughly by an experienced qualitative data analyst to develop an emergent, data-driven codebook. A constant comparative method, which was not based on a grounded-theory (GT) design, was used to discern both common and distinct perspectives by repeat pregnancy status. Instead, we used a saliency analysis approach to assesses both the recurrence and importance of each code, which represented a distinct concept [25, 26]. All data produced were analyzed as opposed to excluding some data because it was not recurring and did not meet the definition of a theme per GT standards [27, 28]. A saliency analysis identifies whether each code is one of four mutually exclusive possibilities: (1) highly important and recurrent; (2) highly important but not recurrent; (3) not highly important but recurrent; (4) not highly important and not recurrent[25].

A standard iterative approach was applied to ensure all relevant text had been coded and that codes were applied consistently [29]. Key concepts that were analytically prominent given both their reoccurrence and emphasis by study participant as well as co-occurring concepts were examined and organized into broader thematic categories, where appropriate, to interpret and synthesize data.

During the coding process, each time a concepts was encountered, we first determined if it was new or recurrent. For recurrent concepts, we compared continually information to specific occurrences in related text, applied the code if appropriate, and refined both our coding approach and the code label and definition where necessary. For a unique concept, we applied a standard code labeled “Unique” so that we could easily retrieve and review the associated text to reassess if it was unique and/or important to better understanding participants’ experiences with and views on sexual reproduction, in particular repeat pregnancy. After coding was completed, concept properties were identified and relationships between codes explored. Lastly, concepts and their relationships were integrated into themes and a coherent explanatory model developed [30]. In this paper, we present findings that were highly important and recurrent as well as highly important but not recurrent in order to better understanding the “perspectival knowledge” of participants derived from their lived experiences” [28] as well as to identify underlying beliefs (social, cultural, religious, economic, gendered, legal, etc.) that influenced women’s fertility desires and intentions. Triangulation for recurring themes was undertaken by comparing coding across data sources (participants) and across interviewers (via the debriefing sessions as well as the NVivo analysis). Member check analysis was not feasible because analysis was undertaken after the main study had been completed. In addition to lack of funds to undertake this task, we had not asked participants to consent to future contact after interview completion.

## Results

### Participant characteristics

Demographics, psychosocial, and behavioral factors from 500 pregnant women at enrollment are shown in Table 1. Overall, median maternal age was 23 years (range 15–43 years) and median gestational age was 34 weeks at enrollment. Ninety-seven percent of the women had attended some level of primary education, and 34% were employed outside their homes. Seventy-five percent of women were married, 13% were single, and 12% were separated, divorced, or widowed. Seventy-six percent of women had pregnancies prior to KiBS enrollment, with a median parity of two. The median number of people in the household was three (range 1–14). All the women indicated they had prior knowledge of FP, while 48% reported prior use of FP.

**Table 1:** Baseline maternal characteristics and behaviors of HIV-infected women in the Kisumu breastfeeding study, Kenya, 2003–2009.

Variable (n = 500)^a^	Number (%)
**Age category, years (n = 500)**	
15–19 years	69 (13.8)
20–24 years	213 (42.6)
25–29 years	137 (27.4)
30–43 years	81 (16.2)
**Highest level of education attended (n = 500)**	
None	13 (2.6)
Primary	302 (60.4)
Secondary	166 (33.2)
College/university	19 (3.8)
**Marital status (n = 500)**	
Married	374 (74.8)
Single	64 (12.8)
Divorced/separated/widowed	62 (12.4)
**Work/job outside home (n = 500)**	170 (34.0)
**Average monthly family income, in Ksh.** ^b^ **(n = 498)**	
<2000	64 (12.9)
2,000–4,999	101 (20.3)
5,000–9,999	60 (12.0)
≥10,000	37 (7.4)
Don’t know	236 (47.4)
**Talked to partner about FP use (n = 494)**	251 (50.4)
**Perception of partner’s feeling about FP use (n = 497)**	
Approve	250 (50.3)
Disapprove	117 (23.5)
Don’t know	130 (26.2)
**Intended to use FP at enrollment (n = 470)**	428 (91.1)
**Prior use of FP (n = 466)**	224 (48.1)
**Number of pregnancies prior to enrollment (n = 498)**	
None	120 (24.1)
At least 1	378 (75.9)
**Husband lives in the compound (n = 497)**	349 (70.2)
**Co-residence with co-wives (n = 498)**	25 (5.0)
**Any relative living in the compound (n = 494)**	435 (88.1)
**Ethnic group (n = 500)**	
Luo	427 (85.4)
Other	73 (14.6)

^a^Sample sizes fluctuate for some variables owing to missing data. Some percentages do not sum to 100% owing to rounding.

^b^The exchange rate was approximately 85 Kenya Shillings (Ksh.) per 1 US dollar (rate varies over study period).

Of 500 women on study, 88 (17.6%) had a repeat pregnancy. Table 2 shows that women in the 30–43 age group were less likely to have a repeat pregnancy compared to women in the 15–19 age group (risk difference (RD): -0.21; 95% confidence interval (CI): -0.33, -0.10). Women who had completed secondary school were less likely to have a repeat pregnancy compared to women who had attended primary school only (RD: -0.08; 95% CI: -0.15, -0.01). Those who had attended college/university were less likely to have a repeat pregnancy than those who had attended primary school only (RD: -0.16; 95% CI: -0.27, -0.05). Those who worked or had job outside their homes were less likely to have a repeat pregnancy compared to those who did not work or did not have job outside their homes (RD: -0.07; 95% CI: -0.14, -0.004). Women who had talked to their partner about FP were less likely to have a repeat pregnancy (RD: -0.08; 95% CI: -0.15, -0.02) while those who had prior use of FP were less likely to have a repeat pregnancy (RD: -0.13; 95% CI: -0.20, -0.07). Women living in the same compound as their husbands were more likely to have a repeat pregnancy compared to those whose husbands were not living in the same compound (RD: 0.08; 95% CI: 0.01, 0.15).

**Table 2:** Single predictor analysis of determinants and experiences of repeat pregnancy among HIV-infected women in the Kisumu breastfeeding study, Kenya, 2003–2009.

Variable name^a^	Repeat Pregnancy m/r^b^ (%)	Risk Difference	95% CI	P-value
**Age category, years (n = 500)**				
15–19	18/69 (26.1)	*Referent*	*Referent*	*Referent*
20–24	46/213 (21.6)	-0.04	-0.16, 0.07	0.45
25–29	20/137 (14.6)	-0.11	-0.23, 0.00	0.06
30–43	4/81 (4.9)	-0.21	-0.33, -0.10	0.0003*
**Highest level of education attended (n = 500)**				
None	3/13 (23.1)	0.02	-0.21, 0.26	0.85
Primary	63/302 (20.9)	*Referent*	*Referent*	*Referent*
Secondary	21/166 (12.7)	-0.08	-0.15, -0.01	0.02*
College/university	1/19 (5.3)	-0.16	-0.27, -0.05	0.01*
**Marital status (n = 500)**				
Married	71/374 (19.0)	*Referent*	*Referent*	*Referent*
Single	10/64 (15.6)	-0.03	-0.13, 0.06	0.50
Divorced/Separated/widowed	7/62 (11.3)	-0.08	-0.17, 0.01	0.09
**Work/job outside home (n = 500)**				
Yes	22/170 (13.0)	-0.07	-0.14, -0.00	0.0496*
No	66/330 (20.0)	*Referent*	*Referent*	*Referent*
**Average monthly family income, in Ksh.** ^c^ **(n = 498)**				
<2000	13/64 (20.3)	*Referent*	*Referent*	*Referent*
2,000–4,999	17/101 (16.8)	-0.03	-0.16, 0.09	0.58
5,000–9,999	11/60 (18.3)	-0.02	-0.16, 0.12	0.78
≥10,000	4/37 (10.8)	-0.10	-0.24, 0.05	0.18
Don’t know	43/236 (18.2)	-0.02	-0.13, 0.09	0.71
**Talked to partner about FP use (n = 494)**				
Yes	34/251 (13.5)	-0.08	-0.15, -0.02	0.02*
No	53/243 (21.8)	*Referent*	*Referent*	*Referent*
**Perception of partner’s feeling about FP use (n = 497)**				
Approve	38/250 (15.2)	-0.09	-0.18, 0.00	0.06
Disapprove	28/117 (23.9)	*Referent*	*Referent*	*Referent*
Don’t know	21/130 (16.2)	-0.08	-0.18, 0.02	0.13
**Intended to use FP at enrollment (n = 470)**				
Yes	76/428 (17.8)	0.01	-0.11, 0.13	0.86
No	7/42 (16.7)	*Referent*	*Referent*	*Referent*
**Prior use of FP (n = 466)**				
Yes	23/224 (10.3)	-0.13	-0.20, -0.07	0.0001*
No	57/242 (23.6)	*Referent*	*Referent*	*Referent*
**Number of pregnancies prior to enrollment (n = 498)**				
None	27/120 (22.5)	*Referent*	*Referent*	*Referent*
At least 1	60/378 (15.9)	0.07	-0.15, 0.02	0.10
**Husband lives in the compound (n = 497)**				
Yes	70/349 (20.1)	0.08	0.01, 0.15	0.04*
No	18/148 (12.2)	*Referent*	*Referent*	*Referent*
**Co-residence with co-wives (n = 498)**				
Yes	6/25 (24.0)	0.07	-0.10, 0.24	0.15
No	82/473 (17.3)	*Referent*	*Referent*	*Referent*
**Any relative living in the compound (n = 494)**				
Yes	79/435 (18.1)	0.05	-0.05, 0.14	0.38
No	8/59 (13.6)	*Referent*	*Referent*	*referent*
**Ethnic group (n = 500)**				
Luo	75/427 (17.6)	-0.002	-0.10, 0.09	0.96
Other	13/73 (17.8)	*Referent*	*Referent*	*Referent*

^a^Sample sizes fluctuate for some variables owing to missing data.

^b^m = number of repeat pregnancies for each row category; r = total number in each row category.

^c^The exchange rate was approximately 85 Kenya Shillings (Ksh.) per 1 US dollar (rate varies over study period).

*P-value ≤ 0.05

Table 3 shows the unadjusted ORs from the simple logistic regression models. The odds of having a repeat pregnancy was significantly smaller among women who were older (25–29 years and 30–43 years), more educated (attended secondary), worked outside of the home, had talked to their partner about FP use, perceived their partner as approving of FP, had prior use of FP and had a husband who did not live in the compound. Table 3 also shows the adjusted ORs for the full or saturated model that included all of the listed variables. None of the tests for multicolliearity were significant. In addition, Table 3 shows the adjusted ORs for the parsimonious or reduced model that was built using stepwise selection. Being in the 30–43 year age group (Adjusted odds ratio (AOR) 0.25; 95% CI: 0.07, 0.87), having talked to partner about FP use (AOR 0.53; 95% CI: 0.29, 0.98), and prior use of FP (AOR 0.45; 95% CI: 0.25, 0.82) were all statistically significantly associated with reduced repeat pregnancy. Living with a husband in the same compound (AOR 2.33; 95% CI: 1.14, 4.75) was significantly associated with increased repeat pregnancy.

**Table 3:** Determinants and experiences of repeat pregnancy: multivariate analysis among HIV-infected women in the Kisumu breastfeeding study, Kenya, 2003–2009.

Variable name	Unadjusted OR (95% CI)	Adjusted OR (95% CI) Full Model^a^	Adjusted OR (95% CI) Reduced Model^b^
**Age category, years**			
15–19	*Referent*	*Referent*	*Referent*
20–24	0.78 (0.42, 1.46)	0.89 (0.40, 1.95)	0.97 (0.46, 2.04)
25–29	0.48 (0.24, 0.99)*	0.80 (0.32, 2.03)	0.84 (0.36, 1.96)
30–43	0.15 (0.05, 0.46)*	0.20 (0.05, 0.75)*	0.25 (0.07, 0.87)*
**Highest level of education attended**			
None	1.14 (0.30, 4.26)	1.59 (0.37, 6.68)	
Primary	*Referent*	*Referent*	
Secondary	0.55 (0.32, 0.94)*	0.80 (0.43, 1.50)	
College/university	0.21 (0.03, 1.61)	0.45 (0.05, 4.00)	
**Work/job outside home**	0.60 (0.35, 1.00)*	0.74 (0.40, 1.39)	
**Average monthly income, in Ksh.** ^c^			
<2000	*Referent*	*Referent*	
2,000–4,999	0.79 (0.36, 1.77)	0.68 (0.26, 1.78)	
5,000–9,999	0.88 (0.36, 2.15)	0.68 (0.23, 2.06)	
≥10,000	0.48 (0.14, 1.58)	0.71 (0.18, 2.83)	
Don’t know	0.87 (0.44, 1.75)	0.67 (0.29, 1.53)	
**Talked to partner about FP use**	0.56 (0.35, 0.90)*	0.55 (0.29, 1.04)	0.53 (0.29, 0.98)*
**Perception of partner's feeling about FP use**			
Approve	0.57 (0.33, 0.99)*	0.59 (0.31, 1.13)	0.57 (0.30, 1.07)
Disapprove	*Referent*	*Referent*	*Referent*
Don’t know	0.61 (0.33, 1.15)	0.45 (0.20, 0.97)*	0.41 (0.19, 1.00)
**Prior use of FP**	0.37 (0.22, 0.63)*	0.47 (0.25, 0.87)*	0.45 (0.25, 0.82)*
**Number of pregnancies prior to enrollment**			
None	*Referent*	*Referent*	
At least one	0.65 (0.39, 1.08)	1.11 (0.56, 2.21)	
**Husband lives in the compound**	1.81 (1.04, 3.17)*	3.39 (1.28,8.95)*	2.33 (1.14, 4.75)*
**Co-residence with co-wives**	1.51 (0.58, 3.89)	2.26 (1.02, 10.40)	
**Any relative living in the compound**	1.41 (0.65, 3.10)	0.48 (0.14, 1.73)	
**Ethnic group**			
Luo	0.98 (0.51, 1.88)	0.85 (0.39, 1.87)	
Other	*Referent*	*Referent*	

^a^Full model: Model includes all variables.

^b^Reduced Model: Model with only variables which were significant in the multiple predictor analysis.

^c^The exchange rate was approximately 85 Kenya Shillings (Ksh.) per 1 US dollar (rate varies over study period).

*P-value ≤ 0.05

### Saliency analysis

Qualitative interviews were completed with 40 women between June 2008 and May 2009, of which 22 (55%) had experienced repeat pregnancy. With the exception of one participant who was in her third trimester, all interviews with those experiencing a repeat pregnancy occurred after delivery of that pregnancy. Similar to the overall KiBS sample, the majority (81%) of the qualitative participants ethnically identified as Luo. Median age for the qualitative study participants was 25 years(range 20 to 33 years) with most women (73%) being married or living as married.

Data comparisons between women with a repeat pregnancy and those without a repeat pregnancy as well as by interviewer did not reveal any discernible differences between the perspectives and the experiences described. Overall, themes and patterns were similar for both groups.

#### Reproductive beliefs, attitudes, and behaviors

Qualitative data analyses showed that reproductive beliefs, attitudes, and behaviors centered on three key themes: (1) modern contraception knowledge and use, (2) having the right number of children to fit one’s desires; and (3) unintended pregnancy. Most qualitative study participants stated they were aware of hormonal and barrier methods of contraception available to them as well as their potential side effects (e.g., menstrual irregularities, gastro-intestinal side effects, leg cramps). Some of the participants expressed concerns that hormonal contraception would ‘ruin their womb’, lead to birth defects in future pregnancies, or interact with their HIV medication. Participants explained that personal concerns about hormonal contraception side effects did not deter them from trying such a method; however, their partner’s mistrust of or concerns about hormonal contraception were cited as reasons for not using hormonal contraception.

Nearly three-quarters (70%) of the qualitative study participants (16 with a repeat pregnancy; 12 without a repeat pregnancy) expressed that they did not want to have more children at the time of their interview. For these women, reasons for not wanting more children included already having reached or surpassed their desired ‘right number’ of children and concerns about the economic burden of feeding and sending additional children to school. Among the 12 women (six with a repeat pregnancy; six without a repeat pregnancy) who desired more children the reason stated was that they had not yet reached their ‘right number’. Others shared they were motivated to have more children because of their partners’ wishes, to fulfill God’s will or to prevent gossip about their family.

To help preserve an HIV-positive woman’s health/well-being and to reduce the risk for vertical transmission, qualitative study participants proposed strategies for preventing or delaying pregnancy as well as approaches to take in the event of a pregnancy. In particular, they recommended that HIV-infected women adhere to birth-spacing practices with intervals of two to five years. For women in situations where avoiding pregnancy was neither feasible nor desirable, women suggested obtaining medical care during pregnancy and labor, and following clinicians’ instructions for a healthy pregnancy (e.g., eat well, get plenty of rest, exercise, make sure to take their antiretroviral medications correctly and consistently).

Regardless of their desire for more children, the majority of women who experienced a repeat pregnancy shared that the pregnancy was unintended. These women either were not actively trying to prevent the pregnancy or their contraception failed (e.g., condom breakage, forgetting to take oral contraceptives). Some women shared that they were unaware that pregnancy could occur so soon after giving birth. Others noted that their partner prevented the use of contraception. One participant, whose response fell into the category of highly important but not recurrent described her unintended pregnancy as follows:

‘It was not my choice…I mean I was using family planning, Depo-Provera. So I was being injected, the one for three months. Now when I was injected, I saw three months ending and I could see I had some changes in me, so when was in [the] KiBS [clinic] I asked and was told to go for a pregnancy test. So when I went it was found that I was pregnant…Some people were telling me that I went for the injection when the days had passed but I just went on the date I was told to return…so I don’t know the reason why [the Depo-Provera did not work]… [The reason I was using the Depo-Provera was] I saw now that I had tested positive, now giving birth every now and then would make me tired and when giving birth the child sometimes is not healthy so I was thinking that if you plan for a period of time is when you will give birth later.’

Another participant emphasized dual contraceptive methods to reduce the chances of contraception failure leading to unintended pregnancy:

‘Okay we find that methods of family planning are not effective. There are some which you can use, and by the end of it you find that you are pregnant. So it is advisable that even if you are using other methods like *sindano* [Depo-Provera] which am using now, but the use of condom has to be there.’

#### The church and gender role expectations

In the qualitative study interviews, women discussed the religious importance of the motherhood role. Although the church community was described as accepting and supporting of infertile women, participants indicated that religious leaders encouraged married congregants to ‘fill the earth’ and childless women were counseled by religious leaders to have faith that God would give them children. Scriptures, in particular the story of Sarah and Abraham, were used to demonstrate how keeping one’s faith could change the fate of a childless couple.

Although many women noted that pastors counseled against premarital and extramarital sex because of the risk for contracting HIV and other diseases, none of the participants were aware of religious teachings or pastoral guidance regarding HIV-infected married women and reproductive decision making. However, some participants noted that religious leaders encouraged pregnant women to test for HIV so that they could avoid transmission of HIV to their infant.

Participants also described the religious community’s expectation of a woman’s role as peacekeeper. They shared that religious leaders urged women to maintain happy marriages by agreeing to their husband’s desires, including desires to father more children. Some participants noted that religious leaders depicted women who refuse to comply with her husbands’ wishes as promoting conflict in the home and consequently weakening their marriage. One participant indicated that her religious leader advised men to take a second wife if the first wife was unable to produce an heir:

‘Women like these they give them advice that they should not disagree with the father of the children, I mean the husband can walk away; they are encouraged that God will bless them and they will get children.…They say that if she does not give birth then the husband can marry another wife.’

#### Influence of familial and societal reproduction beliefs and expectations

In discussing familial and societal reproduction beliefs, participants explained that women were expected to have a child shortly after marriage. While married women without children were described as being well respected, they were also said to be more prone to being mistreated and neglected. Participants who reported experiencing difficulty conceiving shortly after marriage noted that one of the motivations to have a child was a desire to please their in-laws. One woman explained the pressure to please in-laws:

‘There is a woman who told me that the mother-in-law nicknamed her ‘goat’. [The mother in law] was saying that this goat has refused to give birth since I bought it while the goat that was bought after it is giving birth. It is like since you got married you don’t want to give birth while the women who got married after you are giving birth, so for someone who has not given birth there is a problem. So you see when someone is sick and you have no child, like a young woman who has gotten married and finds out that she has the [HIV] virus, it can’t stop her from giving birth because if she doesn’t give birth she will not stay [in the marriage]. So you have to give birth even with the virus so that you can have a baby even if it is only one—at least there’s a child.’

Participants explained that having a child was a culturally accepted way to secure a woman’s place within her husband’s family and helped garner community respect. Women noted that upon marriage a woman must secure a place within her husband’s family and home (created by exogamous marriage). Having a child best positions her to secure such a place. If she fails to produce a child, her husband has the right to force her out of her home or to marry another wife. Many women shared that family and friends were likely to encourage a man with a barren wife to take on another wife who could bear children. For a woman who had fertility problems and was unable to have children, the presence of a fertile co-wife could lead to potential loss of her position or status within the family even if her husband did not separate from her. One woman who did not experience a repeat pregnancy explained:

‘…You will find when I have children and my other co-wife does not have a child, the way I will be treated is different from her and you will find that if something was to be shared out, you will find that I get a bigger portion than her. You will even find that if there is an issue to be discussed in a family, I will be given first priority other than her because they view it that after all she does not have a child.’

Women shared that even if a barren woman was not sent away by her husband, her in-laws could force her to leave her home in the event of the husband’s death. Participants explained that a childless widow is unable to inherit her husband’s home, land, or belongings and may be forced to leave the household compound. In contrast, upon giving birth, in-laws may provide a woman and her husband more land to farm or they may help them construct a larger home for the couple and the child. Children were described as helping to strengthen family ties. Participants indicated that giving gifts of clothes and food for children were demonstrations of love and affection by in-laws.

Despite the emphasis on familial and societal expectation for having children, participants made no mention regarding the actual number of children that a woman is expected to produce; however, one repeat pregnancy participant noted that the pressure to have children continues throughout a woman’s fertile life. She expressed that if she did not continue having children, community gossip about her would result:

‘Yes, you know with my status and having given birth to only two children, people may start saying that so and so’s wife has stayed for long without giving birth, you know issues about children may bring problems and this may make me desire more children…as for me I don’t want a baby. I may take long. I was told you may even take five years, so my desire is to even wait for five years.’

#### Reproductive health communication

Women reported that open communication between partners regarding reproductive health and HIV status was ideal, but also described the challenge of disclosing one’s HIV status or expressing a preference for using contraception given the potential for lack of partner acceptance or conflict. Some participants reported their partners insisted on having unprotected sex despite their emphasis on the importance of using condoms or desire to prevent pregnancy. One participant explained,

‘When I started telling him, he told me that he cannot use those things. He told me that those things hold him tight he cannot use them. I told him when we still had this baby that we use [condoms] to avoid having another pregnancy. But that was not important to him and he told me that he wants to give birth to all his children that is [in] my womb and all that is in him. So it was sometimes unfortunate for me because bedroom matters are not supposed to be discussed loudly so there was nothing I could do.’

## Discussion

KiBS participants received counseling on contraceptive use and prevention of unwanted pregnancies at every follow-up visit. Nevertheless, the incidence of repeat pregnancy in our sample was 17.6%. Similarly high incidence of repeat pregnancies has been described in other studies of HIV-positive women [4, 31], especially among HIV-positive women in Zimbabwe and India [4, 14].

The higher proportion of repeat pregnancy observed among women who live with their husband in the same compound compared to those who do not could be due to increased frequency of sexual intercourse with a partner who is more often present. In our study, a higher proportion of women who were married or were residing with their co-wives or any other relative in the same compound had a repeat pregnancy, although these differences were not statistically significant; however, discussion about the pressure to secure one’s place in the family through childbirth emerged in qualitative interviews. Other studies have found an association between repeat pregnancies among HIV-positive women who do not desire pregnancies and family pressure from partners, in-laws and other relatives [4, 32, 33]. Future studies could benefit by collecting quantitative or qualitative data on the influence that household and other community members have on WLWH’s family planning decisions, including contraceptive use and reproductive intentions.

Studies by Smee et al. (2012) and De La Cruz et al. (2011) showed that the number of children a woman already has is negatively associated with repeat pregnancy, which supports our qualitative findings that women may keep having children until they reach the ‘right’ number [14, 32]. Women in the qualitative study shared that HIV status was unlikely to influence HIV-positive women’s decision for future pregnancies, which is similar to findings of other repeat pregnancy studies among HIV-positive women in Africa [14, 34, 35]. We didn’t examine this aspect in the quantitative study as all women were HIV- positive and on ART. However, as supported by findings from a systematic review, there should be no reason to dissuade WLWH who desires to become pregnant provided they have access to ART and are adequately counseled on mother-to-child transmission prevention [12]. Contraceptive use is important in helping women with birth spacing and birth limiting. In a large survey conducted in Kenya, HIV-positive women had similar odds of using FP as HIV-negative women [36]. However, there was low FP use even among women who did not desire having children [36]. Attitudes towards actual use could be influenced by a partner’s decisions, prior use of FP, their partner’s desire for children, the woman’s concerns about FP side effects, concerns about or experiences with antiretroviral (ARVs) and cotrimoxazole drug interactions with FP, disclosure of HIV status to her partner, and her understanding about fertility postpartum. In a separate KiBS publication [37], having talked to one’s partner about FP was significantly associated with a woman’s intention to use FP. In the quantitative analysis, we found that women who talked to their partners about FP were less likely to have repeat pregnancies; however women in the qualitative study also mentioned how difficult it is to talk to their partners about FP. This suggests that if we can make it easier for women and their partners to communicate about FP then more women may be able to control the number of pregnancies they have.

Women in the qualitative study described the difficulty of disclosing their HIV status to their partners. The disclosure question in the quantitative analysis was not asked directly and data were only collected during follow-up. At baseline, the women were asked if their husband knew their HIV status and there was no significant association between the husband knowing the HIV status of their partners and repeat pregnancy. Similar to our qualitative study findings, a study in India found repeat pregnancies to more likely occur in women who had not disclosed their HIV status to their spouse [4]. In western Kenya, women who had not disclosed their HIV status to anyone had the lowest utilization of PMTCT and maternity services [38]. This highlights the importance of incorporating disclosure and FP counseling in the reproductive education of WLWH to help them negotiate their desired number of pregnancies with their partners. Disclosure of HIV status to spouses could also increase use of condom or other contraceptive use, thereby decreasing the potential for repeat pregnancies among WLWH.

Our quantitative analyses highlight the significant associations of age, prior use of FP, having talked to one’s partner about FP and having one’s husband living in the same compound, with repeat pregnancy. In addition, qualitative study participants indicated that repeat pregnancy intentions were influenced by their partner’s desires to have more children, economic considerations, and their partner’s attitudes towards contraception. The qualitative study findings also pointed to pressure to become pregnant again soon after delivering, which may subside with time as a woman secures her place in the home and/or has already had multiple children. This could partly explain the lower likelihood of repeat pregnancies among the older women in addition to biological infertility. While prior use of FP was associated with reduced repeat pregnancies, the influence that FP counseling has in changing WLWH’s FP behaviors is not clear. Evaluation of FP counseling, changes in reproductive intentions and desires, and actual reproductive behavior would be beneficial.

Other potential determinants of repeat pregnancy which emerged in the qualitative analyses included the influence of religious leaders, cultural expectations of married women, perceptions of WLWH on FP use, and familial ties. The qualitative analyses also corroborated the direction of the differences in the proportions of repeat pregnancies for average monthly income, marital status, and co-residency with co-wife or any other relative. This study highlights the benefits of using qualitative data to enrich and contextualize quantitative results. In addition, findings not captured in quantitative analysis are brought out in the qualitative study that may be examined in future research.

### Limitations

This study has several limitations. Findings may not be generalizable to other settings within and outside of Kenya. Assessing factors associated with repeat pregnancy among KiBS participants was not part of the original study objectives. Examination of this issue was determined to be important when we learned from the referral family planning clinics that women were not keeping their DMPA appointments and also observed a surprisingly high frequency of repeat pregnancies during the 24-month post-partum period. While our exploratory approach does not make it possible to make definitive conclusions about the findings, it does provide valuable insights on contextual information that may be important in developing questionnaire items related pregnancy desires and expectations for HIV positive women as well as issues that may need to be addressed in counseling and clinical management. No discernible differences were noted in information provide by qualitative participants based on repeat pregnancy status. It is possible that beliefs and expectations about fertility and women’s roles, which are likely culturally ingrained, may not necessarily change because of HIV status, especially when treatment options are available. Moreover, the perspectives and experiences of our substudy sample may represent shared or overlapping cultural values and beliefs held by multiple ethnic groups within this geographical setting that may not be the case in setting with greater cultural diversity. KiBS participation may have also influenced the perspective held by our small qualitative sample. By not including WLWH from the broader community, it is unknown if we would discover the same themes and patterns in responses to our qualitative questions. Our qualitative findings are not generalizable beyond our substudy sample. Moreover, given our purposive sampling approach, women who took part in an interview may not be representative of the broader KiBS population. Despite receiving training on qualitative interviewing skills, interviewers may have obtained less robust information than possibly interviewers with either greater experience or those that were not a part of the community.

Quantitative data were limited to baseline; thus, the findings do not take into account trends occurring over the time that women did or did not have repeat pregnancies or those who experienced a repeat pregnancy after exiting the study. In addition, women were asked about the number of pregnancies they had before enrolling in KiBS; however, no data were collected on the number of living children. Given our qualitative finding of having the ‘right number of children’, the number of living children may have been an important predictor of repeat pregnancy in our sample. The association of clinical factors and repeat pregnancy was also not assessed. We only collected information on intentions to use FP at baseline as opposed to actual FP use after delivery of the KiBS baby, which would have been more useful in assessing the association between FP and repeat pregnancy. Data on whether the father of the baby or husband or partner knew the HIV status of the woman before delivery was not available at baseline; hence, its relationship to repeat pregnancy was only explored in the single predictor analysis. We also did not collect data on the partner HIV status and its effect on reproductive choices and use of condom.

Recall bias and social desirability bias may be present since the enrollment questions were based on recall of past events, and the women might have reported to the interviewer what they perceived to be most acceptable. Moreover, questionnaire items were not constructed using input from the target population. Consequently, comparing quantitative findings to qualitative ones may be confounded by participant interpretation or study staff explanation of questionnaire questions and response options. We could also not show any effect of being on ART on the subsequent pregnancy. In the qualitative interview, women were not asked about any effects of being on ART and its effects on their subsequent pregnancy. In addition, women did not freely offer this information. We could also not show an association of being on ART and subsequent pregnancy as all women were initiated on ART.

### Conclusions and implications

Our findings suggest that a complex interplay between economic, demographic, religious, interpersonal, psychosocial, and cultural factors may account for reproductive outcomes, particularly in influencing the occurrences of repeat pregnancies among HIV-infected Kenyan women. Programs addressing the reproductive health of HIV-positive women may require additional attention to culture, including the possibility that reproductive decision making extends beyond individual and couple-based desires and intentions. Moreover, in the context of other considerations, a woman’s HIV status may play a minor role in her reproductive decision making. While HIV research has made significant contributions toward addressing facilitators and barriers present in dyadic partnerships, less attention has been given to the influence of family and religious systems that form integral components of the paired relationship in a cultural setting. Targeted educational messages that focus on birth spacing, birth limiting, and resumption of fertility after giving birth could be beneficial if integrated in reproductive health counseling for WLWH and their partners.

To further address unintended pregnancies effectively, an understanding of women’s culture, the influence of their in-laws, and religion are of central importance. While HIV- positive women may be willing to prevent pregnancy, religious teachings such as accepting their husbands’ reproductive desires could over-ride their personal desires. The inclusion of religious leaders in HIV prevention strategies could potentially foster broader influence, especially if these leaders already play a critical role in advising congregation members to get HIV testing to reduce mother to infant transmission. Even in developed countries such as the United States, private religious practices (e.g. prayer, meditation, watching or listening to religious programs, and reading the Bible or religious material) have associated with desire for a child among HIV-positive women [32]. Expanding HIV reproductive education to include in-laws, religious leaders and programs, and the broader community may be beneficial as part of system thinking approach as a problem solving technique. This study is indicative and not conclusive. A larger study with combined quantitative and qualitative approach could throw light on little understood aspects of HIV status and reproductive choices.

## References

[1] UNAIDS. Global Report: UNAIDS Report on the Global AIDS Epidemic: 2012 Geneva: Joint United Nations Programme on HIV/AIDS, 2012.

[2] UNAIDS. Global report: UNAIDS report on the global AIDS epidemic 2010. Geneva: Joint United Nations Programme on HIV/AIDS, 2010.

[3] Oluoch T, Mohammed I, Bunnell R, Kaiser R, Kim AA, Gichangi A, et al Correlates of HIV infection among sexually active adults in Kenya: A national population-based survey. The open AIDS journal. 2011;5:125–34. Epub 2012/01/19. 10.2174/1874613601105010125 22253668 PMC3257551

[4] Suryavanshi N, Erande A, Pisal H, Shankar AV, Bhosale RA, Bollinger RC, et al Repeated pregnancy among women with known HIV status in Pune, India. AIDS care. 2008;20(9):1111–8. Epub 2008/07/09. 10.1080/09540120701842753 . 18608074

[5] French CE, Cortina-Borja M, Thorne C, Tookey PA. Incidence, patterns, and predictors of repeat pregnancies among HIV-infected women in the United Kingdom and Ireland, 1990–2009. Journal of acquired immune deficiency syndromes. 2012;59(3):287–93. Epub 2012/01/10. 10.1097/QAI.0b013e31823dbeac 22227490 PMC3378493

[6] Homsy J, Bunnell R, Moore D, King R, Malamba S, Nakityo R, et al Reproductive intentions and outcomes among women on antiretroviral therapy in rural Uganda: a prospective cohort study. PloS one. 2009;4(1):e4149 Epub 2009/01/09. 10.1371/journal.pone.0004149 19129911 PMC2612743

[7] Westreich D, Maskew M, Rubel D, MacDonald P, Jaffray I, Majuba P. Incidence of pregnancy after initiation of antiretroviral therapy in South Africa: a retrospective clinical cohort analysis. Infectious diseases in obstetrics and gynecology. 2012;2012:917059 Epub 2012/07/11. 10.1155/2012/917059 22778536 PMC3388336

[8] Schwartz SR, Rees H, Mehta S, Venter WD, Taha TE, Black V. High incidence of unplanned pregnancy after antiretroviral therapy initiation: findings from a prospective cohort study in South Africa. PloS one. 2012;7(4):e36039 Epub 2012/05/05. 10.1371/journal.pone.0036039 22558319 PMC3338622

[9] Cliffe S, Townsend CL, Cortina-Borja M, Newell ML. Fertility intentions of HIV-infected women in the United Kingdom. AIDS care. 2011;23(9):1093–101. Epub 2011/04/12. 10.1080/09540121.2011.554515 . 21480008

[10] Thomas TK, Masaba R, Borkowf CB, Ndivo R, Zeh C, Misore A, et al Triple-antiretroviral prophylaxis to prevent mother-to-child HIV transmission through breastfeeding—the Kisumu Breastfeeding Study, Kenya: a clinical trial. PLoS medicine. 2011;8(3):e1001015 Epub 2011/04/07. 10.1371/journal.pmed.1001015 21468300 PMC3066129

[11] Andia I, Kaida A, Maier M, Guzman D, Emenyonu N, Pepper L, et al Highly active antiretroviral therapy and increased use of contraceptives among HIV-positive women during expanding access to antiretroviral therapy in Mbarara, Uganda. American journal of public health. 2009;99(2):340–7. Epub 2008/12/09. 10.2105/AJPH.2007.129528 19059862 PMC2622797

[12] Calvert C, Ronsmans C. Pregnancy and HIV disease progression: a systematic review and meta-analysis. Tropical medicine & international health: TM & IH. 2015;20(2):122–45. Epub 2014/11/02. 10.1111/tmi.12412 . 25358498

[13] Finocchario-Kessler S, Mabachi N, Dariotis JK, Anderson J, Goggin K, Sweat M. "We weren't using condoms because we were trying to conceive": the need for reproductive counseling for HIV-positive women in clinical care. AIDS patient care and STDs. 2012;26(11):700–7. Epub 2012/10/03. 10.1089/apc.2012.0232 . 23025705 PMC4516957

[14] Smee N, Shetty AK, Stranix-Chibanda L, Chirenje M, Chipato T, Maldonado Y, et al Factors associated with repeat pregnancy among women in an area of high HIV prevalence in Zimbabwe. Women's health issues: official publication of the Jacobs Institute of Women's Health. 2011;21(3):222–9. Epub 2011/03/18. 10.1016/j.whi.2010.11.005 . 21411336

[15] Chi BK, Rasch V, Thi Thuy Hanh N, Gammeltoft T. Pregnancy decision-making among HIV positive women in Northern Vietnam: reconsidering reproductive choice. Anthropology & medicine. 2011;18(3):315–26. Epub 2011/11/09. 10.1080/13648470.2011.615909 . 22060125

[16] Todd CS, Stibich MA, Laher F, Malta MS, Bastos FI, Imbuki K, et al Influence of culture on contraceptive utilization among HIV-positive women in Brazil, Kenya, and South Africa. AIDS and behavior. 2011;15(2):454–68. Epub 2010/11/27. 10.1007/s10461-010-9848-z . 21110078

[17] Sofolahan YA, Airhihenbuwa CO. Childbearing decision making: A qualitative study of women living with HIV/AIDS in Southwest Nigeria. AIDS research and treatment. 2012;2012:478065 Epub 2013/01/16. 10.1155/2012/478065 23320152 PMC3539429

[18] Tweya H, Feldacker C, Breeze E, Jahn A, Haddad LB, Ben-Smith A, et al Incidence of pregnancy among women accessing antiretroviral therapy in urban Malawi: a retrospective cohort study. AIDS and behavior. 2013;17(2):471–8. Epub 2012/02/23. 10.1007/s10461-012-0150-0 . 22354359

[19] Kawale P, Mindry D, Stramotas S, Chilikoh P, Phoya A, Henry K, et al Factors associated with desire for children among HIV-infected women and men: a quantitative and qualitative analysis from Malawi and implications for the delivery of safer conception counseling. AIDS care. 2014;26(6):769–76. Epub 2013/11/07. 10.1080/09540121.2013.855294 ; PubMed Central PMCID: PMCPmc3943633. 24191735 PMC3943633

[20] Hernando V, Alejos B, Alvarez D, Montero M, Perez-Elias MJ, Blanco JR, et al Reproductive desire in women with HIV infection in Spain, associated factors and motivations: a mixed-method study. BMC pregnancy and childbirth. 2014;14:194 Epub 2014/06/07. 10.1186/1471-2393-14-194 ; PubMed Central PMCID: PMCPmc4063425. 24902487 PMC4063425

[21] Zhdanova-Redman E. Kenya—traditions and daily life. Available: http://edhelper.com/ReadingComprehension_Geography_78_1.html2011 [updated 10/5/2013]. Available: http://edhelper.com/ReadingComprehension_Geography_78_1.html.

[22] Ayodo A. Luo. New York City: Rosen Publishing Group; 1996.

[23] Owiti GO. The Luo co-wives of Kenya: Using resistance resources to achieve an empowered quality of life: University of Bergen; 2012.

[24] Kleinbaum David G., Klein Mitchel. Logistic Regression, A Self-Learning Text. Third ed Gail M., Kricherberg K., Samet J.M., Tsiatis A., Wong W., editor. Available: http://www.springer.com/series/2848: Springer; 2010 55 p.

[25] Buetow S. Thematic analysis and its reconceptualization as ‘saliency analysis’. Journal of Health Services Research & Policy. 2010;15(2):123–5. 19762883 10.1258/jhsrp.2009.009081

[26] Guest G, McLellan E. Distinguishing the trees from the forest: Applying cluster analysis to thematic qualitative data. Field Methods. 2003;15(2):186–201.

[27] Fram SM. The Constant Comparative Analysis Method Outside of Grounded Theory. Qualitative Report. 2013;18:1.

[28] O'Connor MK, Netting FE, Thomas ML. Grounded Theory Managing the Challenge for Those Facing Institutional Review Board Oversight. Qualitative Inquiry. 2008;14(1):28–45.

[29] MacQueen KM, McLellan E., Kay K., and Milstein B.,. Codebook development for team-based qualitative analysis. Cultural Anthropology Methods Journal. 1998;10(2):31–6.

[30] Taylor SJ, Bogdan R. Introduction to qualitative research methods: The search for meanings. New York: Wiley; 1984.

[31] Obare F, van der Kwaak A, Birungi H. Factors associated with unintended pregnancy, poor birth outcomes and post-partum contraceptive use among HIV-positive female adolescents in Kenya. BMC women's health. 2012;12:34 Epub 2012/10/09. 10.1186/1472-6874-12-34 23039966 PMC3492047

[32] De La Cruz NG, Davies SL, Stewart KE. Religion, relationships and reproduction: correlates of desire for a child among mothers living with HIV. AIDS and behavior. 2011;15(6):1233–42. Epub 2010/08/18. 10.1007/s10461-010-9788-7 . 20714924

[33] Wanyenze RK, Wagner GJ, Tumwesigye NM, Nannyonga M, Wabwire-Mangen F, Kamya MR. Fertility and contraceptive decision-making and support for HIV infected individuals: client and provider experiences and perceptions at two HIV clinics in Uganda. BMC public health. 2013;13:98 Epub 2013/02/05. 10.1186/1471-2458-13-98 23374175 PMC3568663

[34] Myer L, Morroni C, Rebe K. Prevalence and determinants of fertility intentions of HIV-infected women and men receiving antiretroviral therapy in South Africa. AIDS patient care and STDs. 2007;21(4):278–85. Epub 2007/04/28. 10.1089/apc.2006.0108 . 17461723

[35] Bussmann H, Wester CW, Wester CN, Lekoko B, Okezie O, Thomas AM, et al Pregnancy rates and birth outcomes among women on efavirenz-containing highly active antiretroviral therapy in Botswana. Journal of acquired immune deficiency syndromes. 2007;45(3):269–73. Epub 2007/04/24. 10.1097/QAI.0b013e318050d683 . 17450102

[36] Ngugi EW, Kim AA, Nyoka R, Ng'ang'a L, Mukui I, Ng'eno B, et al Contraceptive practices and fertility desires among HIV-infected and uninfected women in Kenya: results from a nationally representative study. Journal of acquired immune deficiency syndromes. 2014;66 Suppl 1:S75–81. Epub 2014/01/15. 10.1097/qai.0000000000000107 . 24413040 PMC4786173

[37] Akelo V, Girde S, Borkowf CB, Angira F, Achola K, Lando R, et al Attitudes toward family planning among HIV-positive pregnant women enrolled in a prevention of mother-to-child transmission study in Kisumu, Kenya. PloS one. 2013;8(8):e66593 Epub 2013/08/31. 10.1371/journal.pone.0066593 23990868 PMC3753279

[38] Spangler SA, Onono M, Bukusi EA, Cohen CR, Turan JM. HIV-positive status disclosure and use of essential PMTCT and maternal health services in rural Kenya. Journal of acquired immune deficiency syndromes. 2014;67 Suppl 4:S235–42. Epub 2014/12/02. 10.1097/qai.0000000000000376 ; PubMed Central PMCID: PMCPmc4251910. 25436823 PMC4251910

