# Correction: Determinants and Experiences of Repeat Pregnancy among HIV-Positive Kenyan Women—A Mixed-Methods Analysis

**DOI:** 10.1371/journal.pone.0134536

**Published:** 2015-07-29

**Authors:** Victor Akelo, Eleanor McLellan-Lemal, Lauren Toledo, Sonali Girde, Craig B. Borkowf, Laura Ward, Kenneth Ondenge, Richard Ndivo, Shirley L. Lecher, Lisa A. Mills, Timothy K. Thomas

The affiliation for the 10^th^ author is incorrect. Lisa A. Mills is not affiliated with #4, and the correct affiliation should be Emory University, Atlanta, GA, United States of America.
